# Diversity of D-Amino Acid Utilizing Bacteria From Kongsfjorden, Arctic and the Metabolic Pathways for Seven D-Amino Acids

**DOI:** 10.3389/fmicb.2019.02983

**Published:** 2020-01-10

**Authors:** Yang Yu, Jie Yang, Li-Yuan Zheng, Qi Sheng, Chun-Yang Li, Min Wang, Xi-Ying Zhang, Andrew McMinn, Yu-Zhong Zhang, Xiao-Yan Song, Xiu-Lan Chen

**Affiliations:** ^1^State Key Laboratory of Microbial Technology, Institute of Marine Science and Technology, Marine Biotechnology Research Center, Shandong University, Qingdao, China; ^2^College of Marine Life Sciences, Institute for Advanced Ocean Study, Ocean University of China, Qingdao, China; ^3^Institute for Marine and Antarctic Studies, University of Tasmania, Hobart, TAS, Australia; ^4^Laboratory for Marine Biology and Biotechnology, Pilot National Laboratory for Marine Science and Technology, Qingdao, China

**Keywords:** D-amino acids (DAA), DAA metabolism, DAA oxidoreductase/dehydrogenase, DAA transaminase, D-serine ammonia-lyase, D-serine ammonia-lyase DSD1, Asp racemase, marine bacteria

## Abstract

D-amino acids (DAAs) are an important component of the refractory dissolved organic matter pool in the ocean. Microbes play a vital role in promoting the recycling of DAAs in the ocean. However, the diversity of marine DAA-utilizing bacteria and how they metabolize DAAs are seldom studied. Here, by enrichment culture with DAAs as the sole nitrogen source, bacteria of 12 families from three phyla were recovered from surface seawater and sediment from Kongsfjorden, Arctic, and seven DAA-utilizing bacterial strains were isolated. These strains have different DAA-utilizing abilities. Of the seven DAAs used, *Halomonas titanicae* SM1922 and *Pseudoalteromonas neustonica* SM1927 were able to utilize seven and five of them, respectively, while the other strains were able to utilize only one or two. Based on genomic, transcriptional and biochemical analyses, the key genes involved in DAA metabolism in each strain were identified and the metabolic pathways for the seven DAAs in these marine bacteria were identified. Conversion of DAAs into α-keto acids is generally the main pathway in marine DAA-utilizing bacteria, which is performed by several key enzymes, including DAA oxidoreductases/dehydrogenases, D-serine ammonia-lyases, D-serine ammonia-lyase DSD1s and DAA transaminases. In addition, conversion of DAAs into LAAs is another pathway, which is performed by amino acid racemases. Among the identified key enzymes, D-serine ammonia-lyase DSD1 and Asp racemase are first found to be employed by bacteria for DAA utilization. These results shed light on marine DAA-utilizing bacteria and the involved DAA metabolism pathways, offering a better understanding of the DAA recycling in the ocean.

## Introduction

Except for glycine, natural amino acids have D/L-enantiomers. However, living organisms almost exclusively use L-amino acids (LAAs) instead of D-amino acids (DAAs) to build blocks of proteins. Although the biological occurrence of DAAs is rare, their functions in a variety of organisms are still very important. For example, in mammals, D-serine influences the functional plasticity of cerebral circuitry throughout life (Billard, 2012), and free D-Asp is also found in mammals and plays an important role in nervous system development and hormone regulation (Ota et al., 2012). In bacteria, D-Ala and D-Glu are essential components of peptidoglycan in many bacterial cell walls, and some DAAs are reported to modulate peptidoglycan synthesis (Lam et al., 2009). Furthermore, DAAs can prevent biofilm formation and break down existing biofilms in *Bacillus subtilis* (Kolodkin-Gal et al., 2010). In yeasts, D-Ala can be utilized as a sole nitrogen source by *Schizosaccharomyces pombe* (Uo et al., 2001).

D-Amino acids are an important component of the dissolved organic matters (DOM) in the ocean. There, they exist in two forms, either combined or free. Most of the combined DAAs are in the peptide chains of the peptidoglycan in bacterial cell walls. The most common combined DAAs present in bacterial peptidoglycan are D-Ala and D-Glu, although some bacterial peptidoglycan contains other DAAs, such as D-Asp in *Lactococcus* (Veiga et al., 2006) and *Enterococcus* (Bellais et al., 2006), and D-Ser in vancomycin-resistant *Staphylococcus aureus* (De Jonge et al., 2002; Vollmer et al., 2008a). Substantial inputs of DAAs to the DOM pool potentially originate from viral lysis of bacteria and protist grazing on bacteria (Weinbauer, 2004; Jørgensen and Middelboe, 2006). Free DAAs in the ocean can be released from bacterial peptidoglycan decomposition by extracellular enzymes (Vollmer et al., 2008b). In addition, it has been found that free DAAs can be synthesized by marine archaea, microalgae and bacteria (Nagata et al., 1999; Yokoyama et al., 2003; Zhang et al., 2016). Also, some bacteria can release a variety of DAAs into the environment. For example, *Vibrio cholera* mainly secretes D-Met and D-Leu, *Staphylococcus aureus* can secrete D-Ala and D-Leu, *Deinococcus radiodurans* secretes large amounts of D-Ala and D-Thr, and *Bacillus subtilis* secretes D-Tyr and D-Phe (Lam et al., 2009; Zhang et al., 2016). There are only a few reports on the content of free DAAs in the ocean. Jørgensen and Middelboe (2006) reported free D-Ala, D-Glu, D-Asp, and D-Ser, ranging from 0.3 to 62 nmol/L, in Roskilde Fjord, making up 3.6% of the dissolved free amino acids (DFAA). Pedersen et al. (2001) reported that the maximum proportions of four dissolved free DAAs, D-Ala, D-Glu, D-Asp, and D-Ser, were up to 10, 6.2, 18, and 9.0% of DFAA in the pore-water of marine sediment, respectively. Despite these studies, it remains largely unclear how DAAs are recycled in the ocean.

Compared to LAAs, DAAs are not easily utilized by marine organisms. In particular, D-Met, D-Val, and D-Leu have been reported to be more difficult for bacteria to utilize than the four common DAAs, D-Ala, D-Glu, D-Asp, and D-Ser, which are regularly used for the construction of bacterial cell walls (Zhang et al., 2016). Therefore, DAAs are regarded as refractory DOM in the ocean. The presence of DAA-utilizing microbes in oceanic environments has been suggested by activity measurements (Perez et al., 2003), which are the most important force driving DAA recycling. However, an understanding of the species of these DAA-utilizing microbes remains limited. There is currently only one report on an investigation of the diversity of DAA-utilizing microbes. Kubota et al. (2016) isolated 28 DAA-utilizing bacterial strains from deep-sea sediments, which were then phylogenetically assigned to *Alphaproteobacteria*, *Gammaproteobacteria*, and *Bacilli*. In addition to these bacterial strains, more DAA-utilizing microbes can be expected to be identified due to the ubiquity of DAAs in the ocean. Moreover, it is still unclear how marine microbes metabolize DAAs to drive DAA recycling in the ocean.

Although the pathways and key enzymes involved in DAA metabolism in marine bacteria are still unknown, several key enzymes involved in DAA metabolism in terrestrial DAA-utilizing bacteria have been identified. Generally, DAA oxidoreductases/dehydrogenases are the most common enzymes found in terrestrial bacteria for DAA metabolism (Tsukada, 1966; Li and Lu, 2009; He et al., 2014). DAA transaminases, catalyzing the transamination of many DAAs to α-keto acids, are also involved in DAA metabolic pathways in several bacteria (Tanizawa et al., 1989; Lee et al., 2006). Uo et al. (2001) and Moore and Leigh (2005) showed that Ala racemase is involved in the catabolism of D-Ala in the archaeon *Methanococcus maripaludis* and the yeast *Schizosaccharomyces pombe*. D-serine ammonia-lyases, which belong to the fold-type II PLP-dependent enzymes and catalyze the degradation of D-Ser to pyruvate and ammonia, are also found in some D-Ser-utilizing bacteria and serve as a key enzyme for D-Ser metabolism and detoxification (Norregaardmadsen et al., 1995; Korte-Berwanger et al., 2013; Li and Lu, 2016). In addition, D-serine ammonia-lyase DSD1, which belongs to the fold-type III PLP-dependent enzymes, is found in Eukarya and is distinct from classical bacterial D-serine ammonia-lyase. Although the genome of the yeast *Saccharomyces cerevisiae* contains a D-serine ammonia-lyase DSD1 gene, it cannot utilize D-Ser as a nitrogen source (Ito et al., 2008). There has still been no report of D-serine ammonia-lyase DSD1 participating in DAA metabolism in DAA-utilizing bacteria.

In this study, we investigate the diversity of DAAs-utilizing bacteria in the surface waters and sediments of Kongsfjorden, an open glacial fjord located on the west coast of Spitsbergen (12°E, 79°N), which is influenced by the inflow of both warm Atlantic water and glacier meltwater (Hop et al., 2002). We also analyzed the metabolic pathways of seven DAAs in seven bacterial strains by identifying the key genes involved. Our results provide new insights into marine DAAs-utilizing bacteria and marine DAA recycling.

## Materials and Methods

### Location Description and Sample Collection

A total of eight samples were collected from the outer to the inner parts of Kongsfjorden, including six surface sediment samples (K1, 2, 3, 4, 6, 8) and two surface seawater samples (K3, 6) during the Chinese Arctic Yellow River Station Expedition in August, 2017. Information on the sampling sites is shown in Table 1. Sediment samples were collected using a sediment grab sampler and stored in airtight sterile plastic bags at 4°C. Surface seawater samples were filtered through polycarbonate membranes with 0.22-μm pores (Millipore Co., United States). Filtered membranes were stored in sterile tubes (Corning Inc., United States) at 4°C.

**TABLE 1:** Information of the sampling sites.

**Sediment**			
**sampling**	**Depth**	**Surface seawater**	
**sites**	**(m)**	**sampling sites**	**Location**
K1	277	–	78°59.395N, 11°39.118E
K2	312	–	78°58.000N, 11°49.750E
K3	356	K3	78°57.736N, 11°54.237E
K4	117	–	78°55.464N, 12°08.553E
K6	65	K6	78°52.333N, 12°34.694E
K8	99	–	78°57.157N, 12°09.815E

### Enrichment and Screening of DAA-Utilizing Bacteria

Two types of media, medium A and B, were prepared to enrich the DAA-utilizing bacteria. Medium A contained 3% sea salt (Sigma, United States) solution supplemented with 1 mM D-Ala, 1 mM D-Glu, 1 mM D-Asp, 1 mM D-Ser, 50 mM glucose, and 0.2 M phosphate buffer (pH 8.0). Medium B contained 1 mM D-Leu, 1 mM D-Met, 1 mM D-Tyr, 1 mM D-Thr, 1 mM D-Phe and those in medium A. DAAs and LAAs were obtained from Shyuanye Biotechnology Co., Ltd. (Shanghai, China).

To obtain DAA-utilizing bacteria from the sediments, the samples from K1, K2, K3, K4, and K8 sediment sites were mixed together to form a single sample (mixed sediment sample). Filtered membranes of surface seawater or 2 g (wet weight) of sediment samples were incubated in 20 mL sterile artificial seawater at 15°C with shaking at 180 rpm for 4–5 h. Then, 0.2 mL solution from each sample was inoculated into 20 mL of medium A or B in 50 mL beakers, which were incubated at 15°C for 4 days with shaking at 180 rpm. Afterward, 0.2 mL of enrichment culture was inoculated into 20 mL fresh medium, and incubated under the same conditions; this was repeated three times to select the DAA-utilizing bacteria. After selection, the microbial community composition and diversity of each sample were analyzed by Guangdong MAIGENE Technology Co. Ltd.

To isolate the bacterial strains utilizing the DAAs from the enrichment cultures, the enrichment cultures were spread on to screening plates containing a single DAA, D-Asp, D-Ser D-Leu, D-Met, D-Tyr, D-Thr, or D-Phe, as the sole nitrogen source. The plates were then incubated at 15°C for 3–5 days until detectable colonies formed. Morphologically distinct colonies were selected, transferred into 5 ml of the same liquid medium, and incubated at 15°C with shaking at 180 rpm for 4 days. The resulting cultures were then spread onto screening plates and incubated at 15°C. This purification procedure was repeated twice.

### Amplification of the 16S rRNA Genes and Phylogenetic Analysis

Bacterial 16S rRNA genes were amplified by PCR with the primers 27F (5′-AGAGTTTGATCCTGGCTCAG-3′) and 1492R (5′-GGTTACCTTGTTACGACTT-3′). The amplified genes were ligated into pMD-19T cloning vectors (TAKARA) and sequenced by Beijing Genomics Institute (China). Isolates with one or more different bases in their 16S rRNA gene sequences were considered to be different strains. Neighbor-joining trees were constructed using MEGA version 7 (Kumar et al., 2016) with the neighbor-joining method and the Kimura two parameter model.

### Transcriptome Analysis

RNA-seq was carried out for transcriptome analysis of strain SM1926. The strain SM1926 was cultured in 2216E (Becton, Dickinson and Company, United States) medium at 25°C to an OD_600_ of 0.8. The cells in the culture were collected by centrifugation at 4000 × *g* for 10 min at 4°C, washed three times with 3% sea salt solution, and then inoculated into the medium containing 1 mM L-Asp or D-Asp, 50 mM glucose, 3% sea salt (Sigma, United States) and 0.2 M phosphate buffer (pH 8.0). Samples were taken at 0 h and at middle logarithmic growth phase. The cells in each sample were collected by centrifugation at 4000 × *g* and 4°C for 10 min. The resulting pellets were frozen in liquid nitrogen and stored at −80°C. Transcriptome sequencing and analysis were performed by WHBioacme Technology Co. Ltd. Significant differences were indicated by *p–*values <0.05 and an absolute fold-change threshold of >2.0. All the RNA-seq read data have been deposited in NCBI’s sequence read archive (SRA) under project accession number PRJNA554256.

### Real-Time qPCR Analysis

Bacteria were cultured in 2216E medium at 25°C to an OD_600_ of 0.8. The cells in the culture were collected by centrifugation at 4000 × *g* for 10 min at 4°C, washed three times with 3% sea salt solution, and then inoculated into the medium containing a single DAA, 50 mM glucose, 3% sea salt (Sigma, United States) and 0.2 M phosphate buffer (pH 8.0). Bacteria were collected at 0 h, and during early and middle logarithmic growth phase. Total RNA was extracted using the RNeasy^®^ Mini Kit (QIAGEN, United States). The extracted RNA was subsequently reverse-transcribed using the *TransScript* All-in-One First-Strand cDNA Synthesis SuperMix for qPCR (Trans, China) and then qPCR was performed using a Light Cycler II 480 System (Roche, Switzerland) following the instructions of SYBR^®^ Premix Ex Taq^TM^ (TAKARA, Japan). The *recA* gene was used as the reference gene.

### Gene Cloning and Protein Expression and Purification

Genes encoding DAA oxidoreductase/dehydrogenase, DAA transaminase, D-serine ammonia-lyase, D-serine ammonia-lyase DSD1 and Asp racemase were cloned from the genomes of the seven DAA-utilizing strains via PCR and overexpressed in *E. coli* BL21 (DE3) cells using the pET-22b vector that contains a His tag for protein purification. Recombinant *E. coli* strains were cultured at 15°C for 24 h with 0.4 mM isopropyl-β-D-thiogalactopyranoside (IPTG) to induce the production of recombinant proteins. Recombinant proteins (except DAA oxidoreductases/dehydrogenases) were purified with Ni^2+^-NTA resin (Qiagen, Germany), followed by desalination on PD-10 Desalting Columns (GE Health-care, America). Recombinant DAA oxidoreductases/dehydrogenases, which are membrane proteins, were purified by the purification method of membrane protein (Xu et al., 2017). Briefly, recombinant *E. coli* cells were disrupted by high pressure crusher, and then centrifuged at 10000 × *g* for 60 min. The soluble fraction was collected and centrifuged at 160,000 × *g* for 60 min again. After centrifugation, the insoluble fraction, which was used as the membrane fraction was collected and solubilized in 50 mM Tris/HCl buffer (pH 8.0) containing 10 mM NaCl, 1% (v/v) glycerol, and 1.5% Triton X-100. The resultant solution was sonicated for 1 min and then centrifuged at 160,000 × *g* for 60 min. The supernatant was collected and the recombinant DAA oxidoreductases/dehydrogenases in the supernatant were purified with Ni^2+^-NTA resin (Qiagen, Germany), followed by desalination on PD-10 Desalting Columns (GE Healthcare, United States).

### Enzyme Assays

The activity of the enzymes (except Asp racemases) was measured by using the 3-methyl-2-benzothiazolinone hydrazine (MBTH) method, based on detecting the quantity of α-keto acid released from the amino acid substrate (Soda, 1968). Seven DAAs (D-Ser, D-Thr, D-Phe, D-Leu, D-Met, D-Tyr, and D-Asp) were used as substrates. The reaction mixture (300 μL) was composed of 50 mM HEPES-NaOH buffer (pH 7.5), 50 mM DAA and 30 μL enzyme. In addition, the reaction mixture for measuring the activity of DAA oxidoreductase/dehydrogenase contained additional 100 μM flavin adenine dinucleotide (FAD), and that for D-serine ammonia-lyase/D-serine ammonia-lyase DSD1 contained additional 20 μM pyridoxal 5′-phosphate (PLP). The reaction was performed at 30°C for 60 min and terminated by incubation at 96°C for 5 min. After reaction, the mixture was centrifuged at 10000 × *g* for 15 min, and 150 μL of the supernatant was taken and mixed with 75 μL 1 M sodium acetate (pH 5.0) and 75 μL 25 mM MBTH. The mixture was incubated at 50°C for 30 min and then cooled to room temperature. The absorbance of the mixture at 320 nm was measured against a blank, which contained all of the components except the DAA substrate. α-ketoglutaric acid was used as a calibration standard. One unit of enzyme activity was defined as the amount of enzyme that catalyzes DAA to generate 1 nmol α-keto acid per min. The activity of Asp racemase was assayed by the circular dichroism (CD) method (Noda et al., 2005). The reaction mixture (0.5 mL) containing 20 mM Tris/HCl (pH 7.5), 20 mM D-Asp (or D-Ser) and the purified enzyme was incubated at 30°C overnight. The same reaction mixture without the purified enzyme was used as a control. Measurement of CD spectra was carried out in a 0.1 cm-path length cell on a JASCO J-1500 Spectrometer (Japan). The scan rate and bandwidth were set to 500 nm/min and 1.0 nm, respectively. Spectra were recorded between 190 and 250 nm by 0.5 nm carving, and were averaged from three scans.

The draft genome sequences of all seven strains were deposited in the National Center for Biotechnology Information (NCBI) Genome database under project accession number PRJNA554250. Functional annotation of the predicted genes was carried out using BLASTP with the NCBI non-redundant protein database and the Kyoto Encyclopedia of Genes and Genomes (KEGG) protein database (Kanehisa et al., 2012).

## Results

### Enrichment of the DAA-Utilizing Bacteria in Kongsfjorden Sediments and Seawater

**FIGURE 1:**
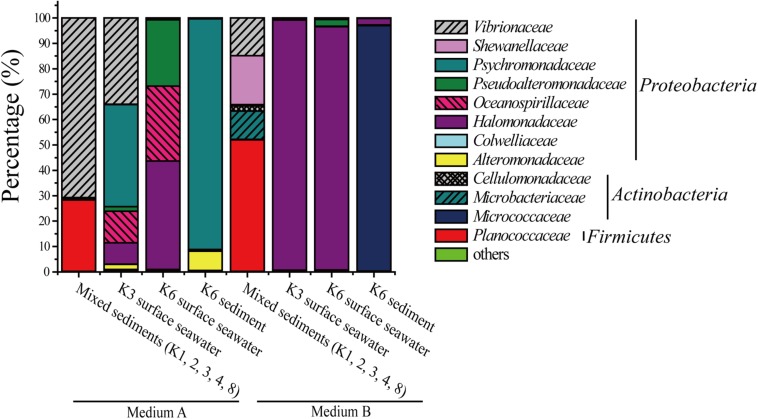
Relative percentage abundances of the phylotypic groups of DAA-utilizing bacteria recovered of four samples from eight sample stations in Kongsfjorden, Arctic.

Two media, A and B, were used to investigate the diversity of DAA-utilizing bacteria in the surface seawater and sediments from Kongsfjorden, Arctic. Medium A, which contained 3% sea salt solution supplemented with 1 mM D-Ala, 1 mM D-Glu, 1 mM D-Asp, 1 mM D-Ser, 50 mM glucose, and 0.2 M phosphate buffer (pH 8.0), was used for screening bacteria that can utilize DAAs present in bacterial peptidoglycan. Medium B, which contained 1 mM D-Leu, 1 mM D-Met, 1 mM D-Tyr, 1 mM D-Thr, and 1 mM D-Phe in addition to those in medium A, was used for screening more DAA-utilizing bacteria in addition to those that can utilize DAAs present in bacterial peptidoglycan. Bacteria were recovered by enrichment culture in these two media, and the microbial community composition of four samples from eight stations were analyzed. A total of 12 bacterial families, falling in three phyla, were found; one family (*Planococcaceae*) in *Firmicutes*, eight families (*Halomonadaceae*, *Psychromonadaceae*, *Vibrionaceae*, *Oceanospirillaceae*, *Pseudoalteromonadaceae*, *Shewanellaceae*, *Alteromonadaceae*, and *Colwelliaceae*) in *Proteobacteria* and three families (*Micrococcaceae*, *Microbacteriaceae*, and *Cellulomonadaceae*) in *Actinobacteria*. Of these, *Halomonadaceae* (31.1%), *Psychromonadaceae* (16.5%), *Vibrionaceae* (15.1%), *Micrococcaceae* (12.2%) and *Planococcaceae* (10.1%) were the most abundant groups (Figure 1). Probably due to the different DAAs in medium A and B, the microbial community composition of all four samples were quite different. For the mixed sediment sample, there was a higher diversity in medium B, which contained 5 families with *Planococcaceae* being dominant, while only *Vibrionaceae* and *Planococcaceae* were present in medium A. In contrast, for the seawater samples, there was a higher diversity in medium A, which included six families recovered from the K3 sample and three from the K6 sample; in medium B, *Halomonadaceae* dominated in both the K3 and K6 samples. *Psychromonadaceae* dominated in the K6 sediment sample in medium A and *Micrococcaceae* dominated in medium B (Figure 1). These results demonstrate the high diversity of DAA-utilizing bacteria in the surface seawater and sediments of Kongsfjorden. Moreover, the results showed that different bacteria were recovered from the same sample in media A and B, which suggests that various DAAs in the media may be utilized by different bacteria. In addition, in the same medium, bacteria recovered from different samples showed different diversity, which suggests that dominant DAA-utilizing bacteria groups may be different in different marine environments.

### Isolation and Identification of the DAA-Utilizing Bacteria

After enrichment culture in media A and B, DAA-utilizing bacteria were further isolated from the enrichment cultures on screening plates with a single DAA, D-Asp, D-Ser, D-Leu, D-Met, D-Tyr, D-Thr, or D-Phe, as the sole nitrogen source (Table 2). These DAAs are reported to be present in marine environments: D-Asp and D-Ser are present in some marine bacterial peptidoglycan and can be released from peptidoglycan decomposition by extracellular enzymes (Bellais et al., 2006; Veiga et al., 2006; Vollmer et al., 2008b); D-Leu, D-Met, D-Tyr, D-Thr, and D-Phe can be released by some marine bacteria (Lam et al., 2009; Zhang et al., 2016). Finally, seven DAA-utilizing bacteria were isolated. Of these, one strain belonged to the genus *Paenarthrobacter* in the phylum *Actinobacteria*, and the other six strains were affiliated with four genera in the class *Gammaproteobacteria*, including *Halomonas*, *Pseudoalteromonas*, *Cobetia*, and *Vibrio* (Table 2). Therefore, these strains belong to the microbial community recovered by enrichment culture. The isolation of these strains from media containing different single DAAs indicates that different strains are likely capable of utilizing different DAAs.

**TABLE 2:** Information of the isolated DAA-utilizing strains.

			**Single DAA**
		**Enrichment**	**screening**
**Strain**	**Isolation station**	**medium**	**plate**
*Halomonas titanicae* SM1922	K3 surface seawater	B	D-Thr/Leu/Met
*Pseudoalteromonas neustonica* SM1927	K6 surface seawater	B	D-Thr/Phe
*Pseudoaltermonas elyakovii* SM1926	K6 surface seawater	A	D-Asp
*Cobetia crustatorum* SM1923	K6 surface seawater	B	D-Ser
*Vibrio atlanticus* SM1925	Mixed sediments sample	A	D-Ser
*Vibrio tasmaniensis* SM1924	Mixed sediments sample	A	D-Ser
*Paenarthrobacter nitroguajacolicus* SM1928	K6 sediment	B	D-Tyr

**FIGURE 2:**
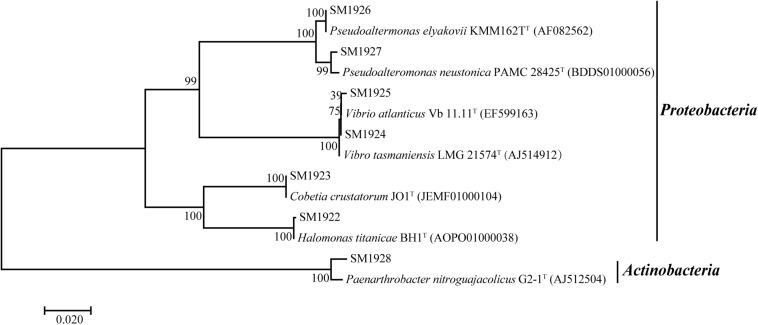
Neighbor-joining phylogenetic tree of the isolated DAA-utilizing bacteria based on the 16S rDNA sequences.

A distance-based neighbor-joining tree was constructed using the sequences of the 16S rRNA genes of the isolated strains and reference sequences from the GenBank database (Figure 2). The closest neighbors of the isolated *Proteobacteria* strains in most cases are from marine sources, while the *Paenarthrobacter* strain SM1928 was closely related to *Paenarthrobacter nitroguajacolicus* G2-1^T^ (AJ512504), which was isolated from forest soil and characterized by the ability to degrade 4-nitroguaiacol (4-NG) (Kotouckova et al., 2004). Except for *Paenarthrobacter nitroguajacolicus* G2-1^T^ (AJ512504) and *Vibrio atlanticus* Vb 11.11^T^ (EF599163) that can utilize D-Ala as a sole carbon source (Kotouckova et al., 2004; Dieguez et al., 2011), none of the closest neighbors in the phylogenetic tree has been previously reported to have DAA-utilizing ability.

### The Ability of the Bacterial Strains to Utilize Different DAAs

**FIGURE 3:**
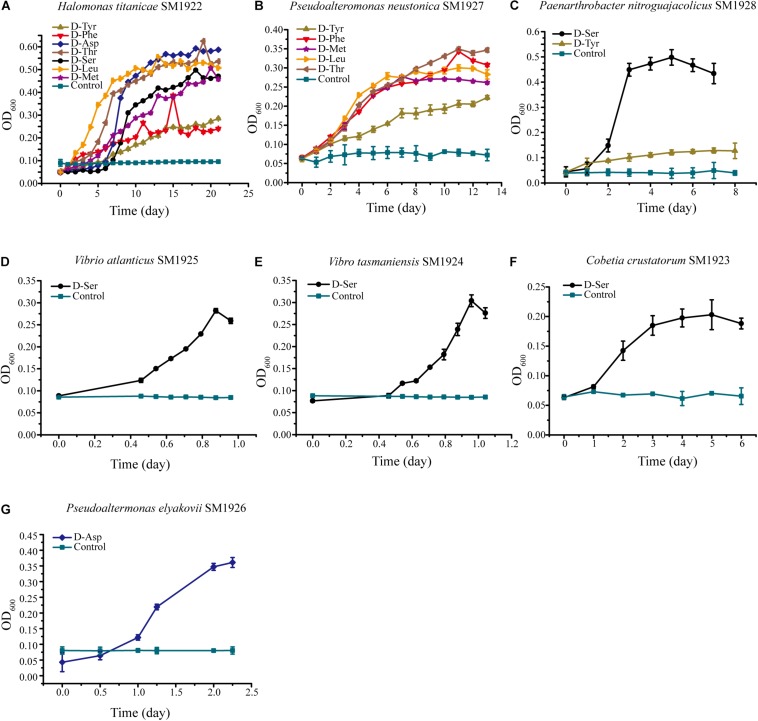
The growth of the seven strains cultured with a single DAA as the sole nitrogen source. The medium contained 1 mM DAA, 50 mM glucose, 3% sea salt (Sigma, United States) and 0.2 M phosphate buffer (pH 8.0). The medium containing all of the components except DAA was used as the control. **(A)** Halomonas titanicae SM1922. **(B)** Pseudoalteromonas neustonica SM1927. **(C)** Paenarthrobacter nitroguajacolicus SM1928. **(D)** Vibrio atlanticus SM1925. **(E)** Vibrio tasmaniensis SM1924. **(F)** Cobetia crustatorum SM1923. **(G)** Pseudoaltermonas elyakovii SM1926.

The ability of the isolated bacterial strains to utilize different DAAs was further investigated. Each strain was cultured in seven different media, each of which contained a single DAA, D-Asp, D-Ser D-Leu, D-Met, D-Tyr, D-Thr, or D-Phe, as the sole nitrogen source. The result showed that three strains had the ability to utilize several kinds of DAAs, although they were all isolated from the plate containing only one kind of DAA. *Halomonas titanicae* SM1922 could utilize all of the seven DAAs as the sole nitrogen source for growth, showing a stronger ability to utilize D-Asp, D-Leu, and D-Thr than the others (Figure 3A). *Pseudoalteromonas neustonica* SM1927 could utilize D-Leu, D-Met, D-Tyr, D-Phe, and D-Thr for growth, with a preference for D-Leu, D-Met, D-Phe, and D-Thr (Figure 3B). The growth of *Paenarthrobacter nitroguajacolicus* SM1928 was strong with D-Ser as the sole nitrogen source, but weak with D-Tyr (Figure 3C). In contrast, the other four strains could only utilize one DAA. *Vibrio atlanticus* SM1925, *Vibrio tasmaniensis* SM1924, *Cobetia crustatorum* SM1923 could utilize D-Ser, and *Pseudoaltermonas elyakovii* SM1926 could utilize D-Asp (Figures 3D–G).

### Prediction of the Key Genes Involved in DAA Metabolism in the Bacterial Strains by Genome Analysis

In order to investigate whether marine bacteria use similar key enzymes in DAA catabolism as terrestrial bacteria, the draft genomes of the seven isolated strains were sequenced and genes encoding DAAs oxidoreductase/dehydrogenase, DAA aminotransferase, D-serine ammonia-lyase or D-serine ammonia-lyase DSD1 were searched for, based on gene annotation. The result showed that, with the exception of the SM1926 strain, in which no such genes were found, all strains contained the predicted putative DAAs oxidoreductase/dehydrogenase genes and some strains also contained predicted D-serine ammonia-lyase genes and/or D-serine ammonia-lyase DSD1 genes (Table 3). The SM1922 strain, which can utilize seven DAAs, has two genes encoding putative FAD-binding oxidoreductase, one gene encoding putative DAA dehydrogenase 3 small subunits, and one gene encoding putative DAA transaminase. Strain SM1928, which can utilize D-Ser and D-Tyr, has two genes encoding putative D-serine ammonia-lyase DSD1 and one gene encoding putative FAD-dependent oxidoreductase. In contrast, Strain SM1927, which can utilize five DAAs, has only one gene encoding putative FAD-binding oxidoreductase. Although the other three strains can utilize only one DAA, their genomes contain different genes that are likely involved in DAA catabolism. Strain SM1923 has two genes encoding putative DAA dehydrogenase, two genes encoding putative FAD-binding oxidoreductase and one gene encoding putative D-serine ammonia-lyase. Strain SM1925 and SM1924 both have one gene encoding putative DAA dehydrogenase small subunit, one gene encoding putative D-serine ammonia-lyase and one gene encoding putative D-serine ammonia-lyase DSD1 (Table 3).

**TABLE 3:** The key genes predicted to be involved in DAA catabolism in the bacterial strains.

**Strains**	**Gene ID**	**Annotation**
***Halomonas titanicae* SM1922**		
	FQP89_15030	FAD-binding oxidoreductase
	FQP89_01690	DAA dehydrogenase 3 small subunits
	FQP89_11185	FAD-binding oxidoreductase
	FQP89_01185	DAA transaminase
***Cobetia crustatorum* SM1923**		
	FQP86_02640	DAA dehydrogenase
	FQP86_09125	FAD-binding oxidoreductase
	FQP86_06845	FAD-binding oxidoreductase
	FQP86_08220	DAA dehydrogenase
	FQP86_13045	D-serine ammonia-lyase
***Pseudoalteromonas neustonica* SM1927**		
	FQP85_04150	FAD-binding oxidoreductase
***Vibrio atlanticus* SM1925**		
	FQP88_12655	DAA dehydrogenase small subunit
	FQP88_16835	D-serine ammonia-lyase
	FQP88_04385	D-serine ammonia-lyase DSD1
***Vibrio tasmaniensis* SM1924**		
	FQP87_22010	DAA dehydrogenase small subunit
	FQP87_04910	D-serine ammonia-lyase
	FQP87_07505	D-serine ammonia-lyase DSD1
***Paenarthrobacter nitroguajacolicus* SM1928**		
	FQP90_21520	FAD-dependent oxidoreductase
	FQP90_13285	D-serine ammonia-lyase DSD1
	FQP90_15205	D-serine ammonia-lyase DSD1

### Transcriptional Analysis of the Predicted Key Genes in the DAA-Utilizing Bacteria by RT-qPCR

**FIGURE 4:**
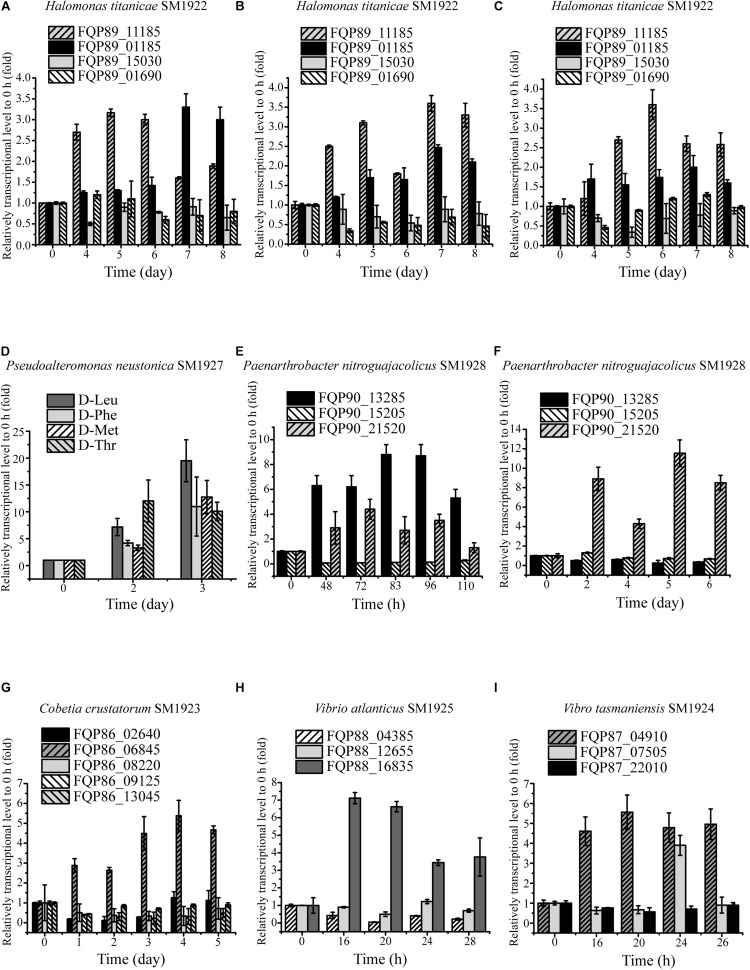
Relative transcriptional levels of the predicted key genes involved in DAA catabolism in the isolated strains analyzed by RT-qPCR. **(A–C)** The relative transcriptional levels of gene FQP89_15030 (FAD-binding oxidoreductase), FQP89_01690 (DAA dehydrogenase 3 small subunit), FQP89_11185 (FAD-binding oxidoreductase) and FQP89_01185 (D-amino-acid transaminase) in strain SM1922 cultured with D-Asp **(A)**, D-Leu **(B),** and D-Thr **(C)** as a sole nitrogen source, respectively. **(D)** The relative transcriptional level of gene FQP85_04150 (FAD-binding oxidoreductase) in strain SM1927 cultured by D-Leu, D-Phe, D-Met, and D-Thr as a sole nitrogen source, respectively. **(E,F)** The relative transcriptional level of gene FQP90_21520 (FAD-dependent oxidoreductase), FQP90_13285 and FQP90_15205 (D-serine ammonia-lyase DSD1) in the strain SM1928 cultured with D-Ser **(E)** and D-Tyr **(F)** as a sole nitrogen source, respectively. **(G)** The relative transcriptional levels of gene FQP86_08220 and FQP86_02640 (DAA dehydrogenase), FQP86_09125 and FQP86_06845 (FAD-binding oxidoreductase) and FQP86_13045 (D-serine ammonia-lyase) in strain SM1923 cultured with D-Ser as a sole nitrogen source. **(H)** The relative transcriptional level of gene FQP88_12655 (DAA dehydrogenase small subunit), FQP88_16835 (D-serine ammonia-lyase) and FQP88_04385 (D-serine ammonia-lyase DSD1) in strain SM1925 cultured with D-Ser as a sole nitrogen source. **(I)** The relative transcriptional levels of gene FQP87_22010 (DAA dehydrogenase small subunit), FQP87_04910 (D-serine ammonia-lyase) and FQP87_07505 (D-serine ammonia-lyase DSD1) in strain SM1924 cultured with D-Ser as a sole nitrogen source.

To investigate whether the strains utilize the predicted key genes shown in Table 3 to metabolize DAAs, the transcription levels of these genes in the six strains grown in the presence of DAA as a sole nitrogen source were analyzed using RT-qPCR, and the results were shown in Table 4. In strain SM1922 that could utilize all seven DAAs, the transcription level of a FAD-binding oxidoreductase gene (FQP89_11185) was significantly up-regulated when the strain was cultured with D-Asp, D-Leu, or D-Thr as the sole nitrogen source, and D-amino-acid transaminase gene (FQP89_01185) was also significantly up-regulated in the late growth phase when the strain was cultured with D-Asp (Figures 4A–C). However, the other FAD-binding oxidoreductase gene (FQP89_15030) or the gene encoding DAA dehydrogenase 3 small subunits (FQP89_01690) was not up-regulated. This suggests that strain SM1922 mainly uses the gene FQP89_11185, encoding a FAD-binding oxidoreductase, to metabolize different DAAs, such as D-Asp, D-Leu, and D-Thr, and the gene FQP89_01185, encoding a D-amino-acid transaminase, is also involved in D-Asp metabolism. Similarly, the transcription level of the only predicted key gene (FQP85_04150), encoding a putative FAD-binding oxidoreductase in strain SM1927, was significantly up-regulated when the strain was cultured with D-Phe, D-Met, D-Leu, or D-Thr as the sole nitrogen source (Figure 4D). This suggests that gene (FQP85_04150) is the key gene in strain SM1927 for the metabolism of DAAs. In contrast, strain SM1928, which could utilize D-Ser and D-Tyr, seems to metabolize these DAAs with different genes. When strain SM1928 was cultured with D-Tyr, the transcription level of gene FQP90_21520, encoding a FAD-dependent oxidoreductase, was significantly up-regulated, but that of gene FQP90_13285 or FQP90_15205 (both encoding D-serine ammonia-lyase DSD1) was not (Figure 4E), suggesting that strain SM1928 may metabolize D-Tyr with an FAD-dependent oxidoreductase as the key enzyme. When strain SM1928 was cultured with D-Ser, the transcription level of gene FQP90_13285 was significantly up-regulated, that of gene FQP90_21520 was up-regulated to a lesser extent and that of gene FQP90_15205 was not up-regulated at all (Figure 4F). This suggests that both D-serine ammonia-lyase DSD1 and FAD-dependent oxidoreductase are involved in D-Ser metabolism in strain SM1928 and that D-serine ammonia-lyase DSD1 may be more important. Although strains SM1923, SM1925, and SM1924 could all only utilize D-Ser as the sole nitrogen source, it seems that they use different enzymes to metabolize D-Ser. When cultured with D-Ser, the transcription level of only a FAD-binding oxidoreductase gene (FQP86_06845) was up-regulated in strain SM1923 (Figure 4G), and that of only a D-serine ammonia-lyase gene (FQP88_16835) up-regulated in strain SM1925 (Figure 4H). However, in strain SM1924, in addition to the significant up-regulation of the transcription level of gene FQP87_04910 (encoding D-serine ammonia-lyase) during its whole growth, gene FQP87_07505 (encoding D-serine ammonia-lyase DSD1) was also up-regulated in the late growth phase (Figure 4I). Taken together, these results demonstrate the diversity of key enzymes involved in DAA metabolism in marine bacteria, suggesting that marine bacteria use different pathways to metabolize DAAs.

**TABLE 4:** Relative transcriptional levels of the predicted key genes involved in DAA catabolism in the isolated strains analyzed by RT-qPCR.

**Strains**	**Gene ID**	**Annotation**	**Fold change in different medium**
SM1922	FQP89_01185	DAA transaminase	3.31 (D-Asp), 2.50 (D-Leu), 2.00 (D-Thr)
	FQP89_01690	DAA dehydrogenase 3 small subunits	1.21 (D-Asp), 0.69 (D-Leu), 1.30 (D-Thr)
	FQP89_11185	FAD-binding oxidoreductase	3.17 (D-Asp), 3.60 (D-Leu), 3.60 (D-Thr)
	FQP89_15030	FAD-binding oxidoreductase	0.91 (D-Asp), 0.89 (D-Leu), 0.80 (D-Thr)
SM1923	FQP86_02640	DAA dehydrogenase	1.26 (D-Ser)
	FQP86_09125	FAD-binding oxidoreductase	0.48 (D-Ser)
	FQP86_06845	FAD-binding oxidoreductase	5.38 (D-Ser)
	FQP86_08220	DAA dehydrogenase	0.71 (D-Ser)
	FQP86_13045	D-serine ammonia-lyase	0.90 (D-Ser)
SM1924	FQP87_22010	DAA dehydrogenase small subunit	0.90 (D-Ser)
	FQP87_04910	D-serine ammonia-lyase	5.56 (D-Ser)
	FQP87_07505	D-serine ammonia-lyase DSD1	3.90 (D-Ser)
SM1925	FQP88_12655	DAA dehydrogenase small subunit	1.22 (D-Ser)
	FQP88_16835	D-serine ammonia-lyase	7.11(D-Ser)
	FQP88_04385	D-serine ammonia-lyase DSD1	0.43 (D-Ser)
SM1927	FQP85_04150	FAD-binding oxidoreductase	19.50 (D-Leu), 10.99 (D-Phe), 12.74 (D-Met), 10.10 (D-Thr)
SM1928	FQP90_21520	FAD-dependent oxidoreductase	4.40 (D-Ser), 11.54 (D-Tyr)
	FQP90_13285	D-serine ammonia-lyase DSD1	8.80 (D-Ser), 0.60 (D-Tyr)
	FQP90_15205	D-serine ammonia-lyase DSD1	0.28 (D-Ser), 1.30 (D-Tyr)

### Identification of the Key Gene Responsible for DAA Metabolism in Strain SM1926 by Transcriptomic Analysis and RT-qPCR

**FIGURE 5:**
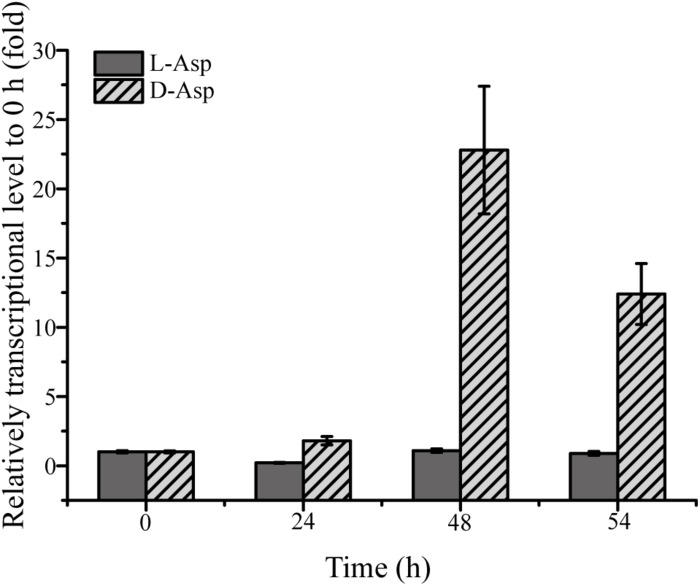
RT-qPCR analysis of the transcriptional level of the putative Asp racemase gene (FQP81_00425) in strain SM1926 cultured with D-Asp as a sole nitrogen source. The *recA* gene was the reference gene.

Because DAA oxidoreductase/dehydrogenase, DAA aminotransferase, D-serine ammonia-lyase or D-serine ammonia-lyase DSD1 were not found in the genome of strain SM1926, based on gene annotation, a transcriptomic analysis was performed on strain SM1926, cultured with L-Asp or D-Asp as the sole nitrogen source, to investigate the key gene responsible for D-Asp metabolism. In strain SM1926 cultured with D-Asp, 1161 genes were up-regulated, of which 144 were up-regulated only in D-Asp but not in L-Asp. In particular, gene FQP81_00425, encoding a putative Asp racemase, was significantly up-regulated in D-Asp (Fold change: 104.4016) but not in L-Asp (Fold change: 0.5860) (Table 5). In addition, adenylosuccinate synthase, argininosuccinate synthase and adenylosuccinate lyase, related to L-Asp metabolism, were up-regulated both in L-Asp and D-Asp. Therefore, it is speculated that the putative Asp racemase, encoded by gene FQP81_00425, is the key enzyme for D-Asp metabolism in strain SM1926, catalyzing the D-to-L racemization of Asp. This was further supported by transcription level analysis by RT-qPCR, which showed that the transcription level of gene FQP81_00425 was significantly up-regulated in D-Asp culture (Fold change: 22), but not in L-Asp culture (Fold change: 0.98) (Figure 5).

**TABLE 5:** Transcriptomic analysis of the genes involved in Asp metabolism in strain SM1926 cultured with L-Asp or D-Asp (1 mM) as a sole nitrogen source.

		**Fold**	
**Gene annotation**	**Medium**	**change**	***p*-value**
Aspartate racemase (FQP81_00425)	L-Asp	0.5860	0.3050
	D-Asp	104.4016	4.8340E−38
Adenylosuccinate synthase (FQP81_10690)	L-Asp	3.2310	0.0002
	D-Asp	2.8540	0.0005
Argininosuccinate synthase (FQP81_07870)	L-Asp	4.1583	0.0001
	D-Asp	2.2701	0.0160
Adenylosuccinate lyase (FQP81_03335)	L-Asp	2.8265	0.0089
	D-Asp	2.6579	0.0024

*The folds were calculated by comparing to 0 h.*

### Transcriptional Analysis of the Gene Encoding Putative Asp Racemase in Other Six Isolated DAA-Utilizing Bacteria

**FIGURE 6:**
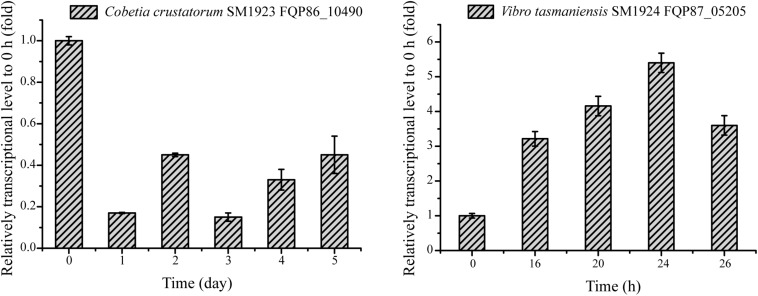
RT-qPCR analysis of the relative transcriptional levels of the genes encoding putative Asp racemase in strains SM1923 and SM1924, cultured with D-Ser as a sole nitrogen source. The *recA* gene was the reference gene.

To investigate whether the homologous genes encoding Asp racemase are involved in DAA metabolism in the other six isolated bacteria, their genomes, with the gene FQP81_00425 from strain SM1926 as a query, were searched and it was found that the genomes of strain SM1923 and SM1924 contain homologous genes of FQP81_00425, i.e., gene FQP86_10490 in strain SM1923 and gene FQP87_05205 in strain SM1924. Since both strain SM1923 and SM1924 can only utilize D-Ser, the transcription levels of these homologous Asp racemase genes in strains SM1923 and SM1924, cultured with D-Ser as a sole nitrogen source, were investigated using RT-qPCR. Gene FQP87_05205 in strain SM1924 was up-regulated but the gene FQP86_10490 in strain SM1923 was not (Figure 6). This suggests that in addition to the D-serine ammonia-lyase gene FQP87_04910 and the D-serine ammonia-lyase DSD1 gene FQP87_07505 (Figure 4I), gene FQP87_05205, which encodes a homolog of Asp racemase, may be involved in D-Ser metabolism in strain SM1924.

### Analysis of the Activity of the Predicted Key Enzymes Involved in DAA Metabolism

In order to further identify the predicted key enzyme genes involved in DAA metabolism, we tried to express these genes and to analyze their activity. We expressed eleven genes whose transcription levels were up-regulated (Table 4) in *E. coli* BL21 (DE3) cells. Finally, nine genes were successfully expressed, and the recombinant proteins were purified, but the expression of the other two genes (FQP86_06845 from SM1923 and FQP85_04150 from SM1927) encoding two membrane proteins failed. The purified recombinant proteins included seven intracellular proteins and two membrane proteins, these are, DAA transaminase TRA-01185 (FQP89_01185) from SM1922, D-serine ammonia-lyases DSD-16835 (FQP88_16835) from SM1925 and DSD-04910 (FQP87_04910) from SM1924, D-serine ammonia-lyase DSD1s DSD1-13285 (FQP90_13285) from SM1928 and DSD1-07505 (FQP87_07505) from SM1924, and Asp racemases AspR-00425 (FQP81_00425) from SM1926 and AspR-05205 (FQP87_05205) from SM1924, and two membrane proteins, DAA oxidoreductases/dehydrogenases DAO-11185 (FQP89_11185) from SM1922 and DAO-21520 (FQP90_21520) from SM1928. Then, the DAA deamination activities of the recombinant DAA transaminase, D-serine ammonia-lyases, D-serine ammonia-lyase DSD1s were examined using a spectrometric method, and the activity of the recombinant Asp racemases was assayed using a circular dichroism (CD) method.

**FIGURE 7:**
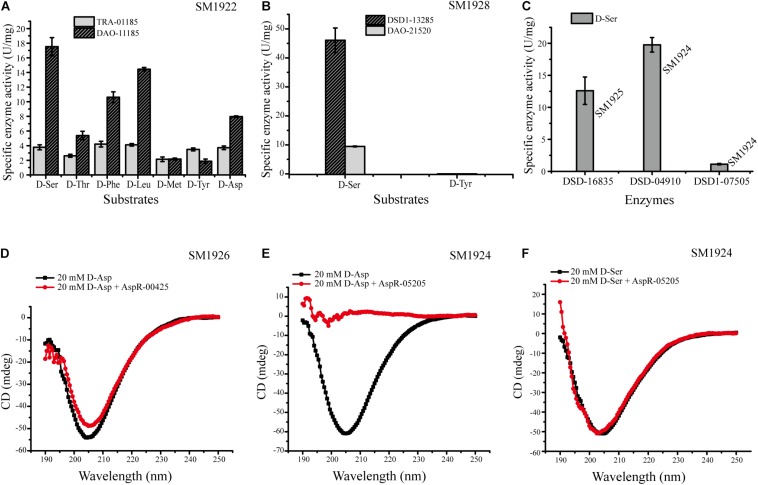
Activities of the recombinant enzymes from strains SM1922, SM1924, SM1925, SM1926, and SM1928. **(A)** Activities of DAO-11185 and TRA-01185 from strain SM1922 against seven DAAs. **(B)** Activities of DAO-21520 and DSD1-13285 from strain SM1928 against D-Ser and D-Tyr. **(C)** Activities of DSD-16835 from strain SM1925 and DSD-04910/DSD1-07505 from strain SM1924 against D-Ser. **(D)** Detection of the Asp racemization activity of AspR-00425 from strain SM1926. **(E,F)** Detection of the Asp racemization activity **(E)** and the ser racemization activity **(F)** of AspR-05205 from strain SM1924.

The enzymes DAO-11185 and TRA-01185 from strain SM1922 all had deamination activity against each of the seven DAAs utilized by the strain (Figure 7A), which further suggests that these enzymes are key enzymes involved in the metabolism of the seven DAAs in this strain. DAO-21520 and DSD1-13285 from strain SM1928 both had deamination activity against D-Ser (Figure 7B), supporting the conclusion that they are key enzymes involved in the metabolism of D-Ser in this strain. However, although the transcription level of gene FQP90_21520 was up-regulated in strain SM1928 cultured with D-Tyr, the *in vitro* deamination activity of DAO-21520 against D-Tyr was not detected. For strain SM1924 that can utilize only D-Ser, DSD-04910 and DSD1-07505 both had deamination activity against D-Ser, although the ability of DSD-04910 to deaminate D-Ser was much higher than that of DSD1-07505 (Figure 7C), suggesting that both of these enzymes are likely involved in the metabolism of D-Ser in this strain, and that DSD-04910 is likely the key enzyme, consistent with the result of transcriptional analysis (Table 4). In addition, we found that the putative D-Asp racemase AspR-05205 from strain SM1924 showed high D-Asp racemization activity but no activity against D-Ser *in vitro* (Figures 7E,F), although the transcription level of its gene FQP87_05205 was up-regulated when the strain was cultured with D-Ser. Thus, the function of gene FQP87_05205 in strain SM1924 in D-Ser metabolism needs further study. For strain SM1925, only FQP88_16835 (putative D-serine ammonia-lyase) was up-regulated when it was cultured with D-Ser, and recombinant DSD-16835 showed deamination activity against D-Ser (Figure 7C), indicating that DSD-16835 is the key enzyme in the metabolism of D-Ser in this strain. For strain SM1926, the transcription level of FQP81_00425 (putative Asp racemase) was up-regulated when it was cultured with D-Asp, and AspR-00425 showed D-Asp racemization activity (Figure 7D), indicating that AspR-00425 is the key enzyme in the metabolism of D-Asp in this strain. Taken together, these results demonstrated the function of a majority of the predicted key enzymes in the isolated strains by *in vitro* activity analysis, which further support the conclusion that these enzymes are key enzymes involved in the metabolism of DAAs in these strains.

## Discussion

Although there is a considerable amount of DAAs derived from bacterial peptidoglycan and released by microorganisms in the ocean, only a few DAA-utilizing bacteria have been reported. Kubota et al. (2016) reported 28 marine DAA-utilizing bacterial strains from the classes *Alphaproteobacteria*, *Gammaproteobacteria* and *Bacilli.* The results presented herein indicate that there are likely a larger number of DAA-utilizing bacteria in the ocean. Using enrichment cultures with DAAs as the sole nitrogen source, it was found that potential DAA-utilizing bacteria, recovered from both surface seawater and surface sediments of Kongsfjorden, belong to 12 bacterial families from three phyla. Moreover, seven DAA-utilizing bacterial strains were isolated, which have not previously been reported to utilize DAAs except that the closest neighbor of strain SM1928 and SM1925, *Paenarthrobacter nitroguajacolicus* G2-1^T^ (AJ512504) and *Vibrio atlanticus* Vb 11.11^T^ (EF599163), were reported to be able to utilize D-Ala as their sole carbon source. Furthermore, it was found that these bacterial strains are quite different in their ability to utilize various DAAs. Among the seven DAAs used, some strains could utilize several DAAs, whereas some strains could only utilize a single DAA. In summary, the results presented herein show that there are diverse DAA-utilizing bacteria in the ocean and that each DAA can be utilized by a certain group of bacteria, which, however, still needs further study.

**FIGURE 8:**
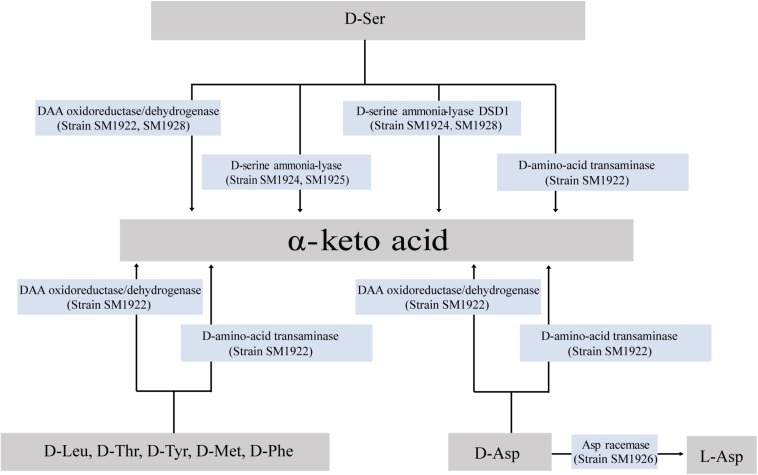
Pathways for D-Leu, D-Met, D-Phe, D-Thr, D-Tyr, D-Ser, and D-Asp metabolism in the isolated marine bacteria.

Although a small number of DAA-utilizing bacteria have been found in the ocean, the pathways for bacterial DAA metabolism remain unknown. In this study, DAA metabolic pathways were investigated by identifying the key enzyme genes involved in DAA metabolism in the isolated bacterial strains through transcriptional and biochemical analyses. In summary, DAA oxidoreductases/dehydrogenases, which catalyze DAAs into α-keto acids, were found to be widely used enzymes for DAA metabolism in marine bacteria (Figure 8) and all of the seven used DAAs (D-Asp, D-Ser, D-Leu, D-Met, D-Tyr, D-Thr, and D-Phe) could be metabolized via DAA oxidoreductases/dehydrogenases (in strain SM1922 for all seven DAAs and strain SM1928 for D-Ser). Furthermore, DAA transaminase is also likely used by strain SM1922 to catalyze the deamination of the seven DAAs to α-keto acids. These results are consistent with the findings for terrestrial bacteria (Wild et al., 1974; Tanizawa et al., 1989; Tanigawa et al., 2010; He et al., 2014; Xu et al., 2017). In addition to the pathways involved in DAA oxidoreductases/dehydrogenases and DAA transaminase, D-Ser can be metabolized via two other enzymes, which can be converted into α-keto acid by D-serine ammonia-lyase (strain SM1925 and SM1924) or by D-serine ammonia-lyase DSD1 (strain SM1928 and SM1924); D-Asp can be metabolized via Asp racemase, and converted into L-Asp (strain SM1926). Therefore, the conversion of DAAs into α-keto acids is generally the main pathway in marine DAA-utilizing bacteria, which is performed by several key enzymes, including DAA oxidoreductases/dehydrogenases, D-serine ammonia-lyases, D-serine ammonia-lyase DSD1s and DAA transaminases. Conversion of DAAs into LAAs is an additional pathway, which is performed by amino acid racemases. Of the identified key enzymes involved in DAA metabolism in marine bacteria, while DAA oxidoreductase/dehydrogenase, DAA transaminase and D-serine ammonia-lyase have been reported to be employed in terrestrial DAA-utilizing bacteria, D-serine ammonia-lyase DSD1 and Asp racemase are found here to be employed by DAA-utilizing bacteria for DAA metabolism. D-serine ammonia-lyase DSD1 has so far only been reported to be involved in detoxification against D-Ser in *Saccharomyces cerevisiae* (Ito et al., 2008), and Asp racemase is only reported to be involved in endogenous D-Asp biosynthesis in bacteria, archaea and mammals (Johnston and Diven, 1969; Dunlop et al., 1986; Yohda et al., 1996).

Although the transcription levels of genes FQP86_06845 from SM1923 and FQP85_04150 from SM1927, which encode putative oxidoreductases/dehydrogenases, were found to be up-regulated when the strains utilized DAAs, the functions of these genes were unable to be identified due to the failure of heterogeneous expression. Therefore, the DAA metabolism pathways in these strains still needs further study. In addition to the seven DAAs investigated in this study, bacteria utilizing other DAAs in the ocean and their metabolic pathways still need to be investigated. Our results and similar studies have an important significance for revealing the recycling of refractory free DAAs in the ocean.

## Data Availability Statement

The datasets generated for this study can be found in the National Center for Biotechnology Information (NCBI) Genome database/PRJNA554250/, https://www.ncbi.nlm.nih.gov/bioproject/PRJNA554250.

## Author Contributions

YY and JY performed the all experiments. L-YZ and QS helped in experiments. C-YL collected the samples. Y-ZZ designed the study. X-LC, X-YS, and X-YZ directed the study. YY and X-LC wrote the manuscript. AM and MW edited the manuscript.

## Conflict of Interest

The authors declare that the research was conducted in the absence of any commercial or financial relationships that could be construed as a potential conflict of interest.
